# Systemic Analysis of RNA Alternative Splicing Signals Related to the Prognosis for Head and Neck Squamous Cell Carcinoma

**DOI:** 10.3389/fonc.2020.00087

**Published:** 2020-02-07

**Authors:** Zhexuan Li, Xun Chen, Ming Wei, Guancheng Liu, Yongquan Tian, Xin Zhang, Gangcai Zhu, Changhan Chen, Jiangyi Liu, Tiansheng Wang, Gongbiao Lin, Juncheng Wang, Gengming Cai, Yunxia Lv

**Affiliations:** ^1^Department of Otolaryngology Head and Neck Surgery, Xiangya Hospital, Central South University, Changsha, China; ^2^Department of Stomatology, First Affiliated Hospital of Quanzhou, Fujian Medical University, Quanzhou, China; ^3^Department of Otolaryngology Head and Neck Surgery, Affiliated Hospital of Guilin Medical University, Guilin, China; ^4^Department of Otolaryngology Head and Neck Surgery, The Second Xiangya Hospital, Central South University, Changsha, China; ^5^Quanzhou Disease Prevention and Control Center, Quanzhou, China; ^6^Department of Otolaryngology Head and Neck Surgery, The Third Xiangya Hospital, Central South University, Changsha, China; ^7^Department of Otolaryngology Head and Neck Surgery, First Affiliated Hospital of Quanzhou, Fujian Medical University, Quanzhou, China; ^8^Department of Thyroid Surgery, The Second Affiliated Hospital of Nanchang University, Nanchang, China

**Keywords:** head and neck squamous cell carcinoma (HNSCC), TCGA database, prognosis, Alternative Splicing (AS), oncology

## Abstract

Alternative splicing (AS) is an important mechanism that is responsible for the production of protein diversity. An increasing body of evidence has suggested that out-of-control AS is closely related to the genesis and development of cancer. Systematic analysis of genome-wide AS in head and neck squamous cell carcinoma (HNSCC) has not yet been carried out, and consideration of this topic remains at the preliminary stage and requires further investigation. In this study, systemic bioinformatic analysis was carried out on the genome-wide AS events of 555 clinical HNSCC samples from the TCGA database. Firstly, we statistically analyzed the distributions of seven AS event types in HNSCC samples. Then, through univariate survival analysis, we observed the relationship between AS and the prognosis of the disease and found that 437 intersections of AS events were significantly related to overall survival. Among them, 335 cross-genes showed a high degree of consistency in the genes associated with overall survival and recurrence. The overall survival was significantly related to AS events. Besides, the frequency of overall survival-related ES events was evidently reduced, while the AP and the AT events were increased. In addition, AT events accounted for the largest proportion. Further, multiple regression model analysis proved that AS could become a new classification method for HNSCC, and KEGG enrichment analysis proved that most genes and proteins interacting with AS events had different biological functions and were associated with a variety of diseases. Finally, through the selection of characteristic HNSCC genes and the construction of a prognostic model, seven cross-genes related to survival and recurrence were screened out, and these characteristic genes were verified by multivariate survival model analysis so as to classify the prognosis at different splicing times and gene expression levels. These results have laid a solid foundation for our further research and play a decisive role in showing the correlation of AS with the prognosis of HNSCC.

## Introduction

HNSCC is a kind of head and neck malignant tumor that is commonly seen in the clinic, accounting for about 5–10% of systemic malignant tumors, with an average morbidity of about 10-5/100,000 (1). In recent years, the morbidity of such tumors with high malignant grade shows an increasing trend. Clinically advanced cases make up about 50%, despite the rapid development of medical technology, as well as the increasingly improved early diagnosis technique of HNSCC (2–4). Over the past 20 years, great achievements have been made in the surgical method (5), radiotherapy (6), and chemotherapy (7); however, the 5-years survival rate for HNSCC, especially for the advanced patients, has not yet been remarkably improved (8). This ineffectiveness of current therapies underscores the urgent need for new discoveries leading to more effective treatment pathways.

The cancer genome atlas (TCGA) is a collection of genomic information about a variety of tumor types, including GBM, designed to classify and identify genomic changes that cause cancer in order to create a comprehensive cancer genome atlas (9). In the past 10 years, the TCGA project has generated a lot of genomics and proteomics data; these data represent the 33 types of cancer in more than 11,000 tumor molecular structures, mainly concentrated on the assessment of somatic non-synonymous protein change mutation and the mutation of gene expression (10, 11). In this paper, access to and download from the TCGA database and global gene expression profile analysis and database mining were conducted to find the potential association between genes and the overall survival rate of multiple malignancies.

Protein diversity is crucial for the obvious regulatory and functional complexities of eukaryocytes. Pre-mRNA AS is a general mechanism that uses a limited set of genes to produce mRNA isomers (12). AS is a process in which the introns of most human multi-exon genes are deleted and specific exons are alternatively included or excluded (13, 14). Apart from protein diversity, the mRNA isomer translation level can also be down-regulated by introducing AS, leading to degradation of the early termination codon (15). Therefore, AS is an important process; changes in the splicing pattern are closely correlated with protein functions, and it is involved in multiple human physiological functions, such as hematopoiesis (16), brain development (17), and muscular activity (18). Differences in the gene expression levels of splicing regulators have been observed in many cancers, and these proteins generally influence the splicing patterns of many genes that play a role in certain cancer-specific biological pathways, including cell cycle progression, cell proliferation and migration, and RNA processing. The prevalence of AS is currently estimated at the genome-wide level using high-throughput methods, including microarrays, expressed sequence tag analysis, and sequencing (13). The AS process can explain the difference between estimates of the number of genes and the number of proteins.

The protein diversity of cancer genome analysis is the key to the regulation and functional complexity of eukaryotic cells. Individual changes in regulatory binding sites or changes in protein-coding sequences may have strong functional effects (10). However, an increasing number of recent studies have demonstrated that AS is also markedly correlated with cancer genesis and development (19, 20). Out-of-control AS participates in multiple carcinogenic processes, including proliferation (21), apoptosis suppression (22), angiogenesis (23), immune escape (21), and metastasis (24). For example, exon-skipping events in MST1R are controlled by SF2/ASF through AS of the RON proto-oncogene (25). Convergence of Acquired Mutations and Alternative Splicing of CD19 lead to the activity of splicing factor SRSF3 was destroyed, AS also affects the immunotherapy of leukemia (26). Met exon 14 was observed in some lung cancer patients, resulting in a deletion of the protein region that inhibits its kinase catalytic activity (27). More recently, analyses of AS have also shown prognostic value for a variety of cancer types, including non-small cell lung cancer (15), ovarian cancer (28), breast cancer (29), uveal melanoma (30), and glioblastoma (31). However, the alternative splice associated with survival in head and neck squamous cell carcinoma (HNSCC) has not been extensively studied. Nonetheless, few studies on head and neck squamous cell carcinoma (HNSCC) have been reported, and research on the subject remains at the preliminary stage (19). This paper aimed to carry out systemic bioinformatic analysis of the HNSCC clinical samples from the TCGA database and open up a novel path for exploring the pathogenesis, prognosis biomarkers, and therapeutic targets of HNSCC from the point of view of AS, thus more accurately guiding clinical treatment and judging prognosis.

## Materials and Methods

### Data Downloading, Preprocessing and Overall Analysis Process

**Figure 1:**
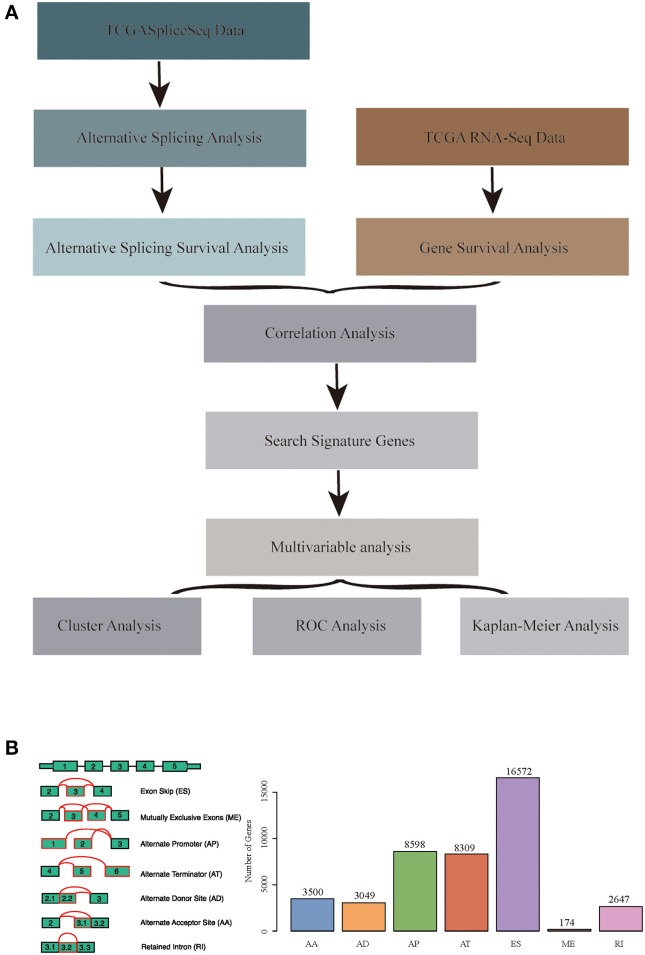
**(A)** Schematic diagram of the research methodology. **(B)** Overview of seven types of alternative splicing (AS) events in this study. Illustrations for seven types of AS events: ES, Exon Skip; ME, Mutually Exclusive Exons; RI, Retained Intron; AP, Alternate Promoter; AT, Alternate Terminator; AD, Alternate Donor site; AA, Alternate Acceptor site.

RNA-seq AS event data for HNSCC were obtained from the TCGASpliceSeq database (32), which covered a total of 555 samples, including 43 normal samples. Additionally, the RNA-seq expression profile data of HNSCC and para-carcinoma were obtained from the TCGA database (33), which involved 546 samples, including 44 normal samples. Moreover, all clinical follow-up data (including 527 samples) were downloaded from the TCGA database. The data processing and statistical modeling are crucial and vital, as the information extracted from the raw data will depend on these steps. A better understanding of data processing and statistical algorithms and methods are important to achieve statistically relevant and optimal biological information. In order to obtain data with high accuracy, integrity, and consistency, we pre-processed TCGA data (34). The data were pre-processed as follows: the RNA-Seq expression profile FPKM dataset was downloaded and further converted into TPM data; at the same time, the ID was transformed using the genome file of GENCODE (GRCh38.p2) (35), and the protein-encoding genes were obtained. A total of 498 common samples in TCGASpliceSeq and RNA-Seq were then enrolled in this study, and a total of 19,754 genes with expression values were obtained as the total gene set for this study. The overall analysis process is presented in Figure 1A.

### Analysis of mRNA AS Events in HNSCC Patients

The TCGASpliceSeq database had analyzed the mRNA splicing pattern using SpliceSeq tool (36) based on TCGA RNA-Seq data. There were seven types of AS events, including Exon Skip (ES), Mutually Exclusive Exons (ME), Retained Intron (RI), Alternate Promoter (AP), Alternate Terminator (AT), Alternate Donor site (AD), and Alternate Acceptor site (AA). In addition, the distribution of all coding genes among these seven different types of data was analyzed in the HNSCC samples.

### Screening of Survival-Related AS Events

Differences in the AS of genes may result in gene diversity, while changes in the gene expression level would affect patient survival. Survival analysis is a method for analyzing and deducing the survival time of an organism or a person based on the data obtained from experiment or investigation so as to examine the relationships of survival time and outcome with numerous influence factors as well as their degrees. It is also referred to as survival rate analysis.

In this study, the different gene-splicing events obtained from disease samples were subjected to univariate survival analysis using the “survival” package in R (37). Genes satisfying the significant level of *p* < 0.05 were selected as the prognosis differential AS events.

### Analysis of AS Event Types of Prognosis-Related Genes

AS can affect the protein diversity translated by genes. In this study, the prognosis-related AS events were selected, and the gene distribution among these genes was determined so as to analyze the gene distribution among the various types of prognosis-related AS events.

### Analysis of the Prognosis Factors of HNSCC AS Events

To observe whether AS events could serve as prognosis factors, the 20 most significant genes among the AS types were selected for multivariate regression model analysis so as to observe their classification of prognosis.

### Construction of a Gene Interaction Network for various Types of AS Events That Were Markedly Correlated With Prognosis

To observe the gene associations among the various types of AS events that were markedly correlated with prognosis, these genes were mapped to the String database (38), respectively. Then, the interactions of these genes were obtained using score >0.4, and Cytoscope was used for visualization.

### Analysis of Gene Functions in Various Types of AS Events That Were Markedly Correlated With Prognosis

To observe the gene functions in various types of AS events that were markedly correlated with prognosis, KEGG enrichment analysis was carried out on the AS genes evidently correlated with prognosis in each type using the “clusterprofile” package in R, so as to observe the enrichment pathways of these genes.

### Correlation Analysis Between Gene Expression Profile and Prognosis in AS Events That Were Markedly Correlated With Prognosis

TCGA RNA-Seq expression profile data were used for univariate survival analysis of each gene to observe the relationships between gene expression and prognosis in AS events that were markedly correlated with prognosis; in addition, the influence of the expression profiles of genes involved in AS events on prognosis was also examined.

### Selection of HNSCC Feature Genes

Genes with a Pearson correlation coefficient between gene expression and AS events of >0.2 or <-0.2 were selected as the prognosis feature genes.

### Construction of the HNSCC Prognosis Model

To construct the prognosis-predicting indexes that were suitable for HNSCC patients and to facilitate clinical practice, the prognosis feature genes were selected to construct the multivariate survival model, so as to observe the classification of prognosis by these prognosis feature genes at AS event and expression profile levels.

## Results

### Analysis of mRNA AS Events in HNSCC Patients

**Figure 2:**
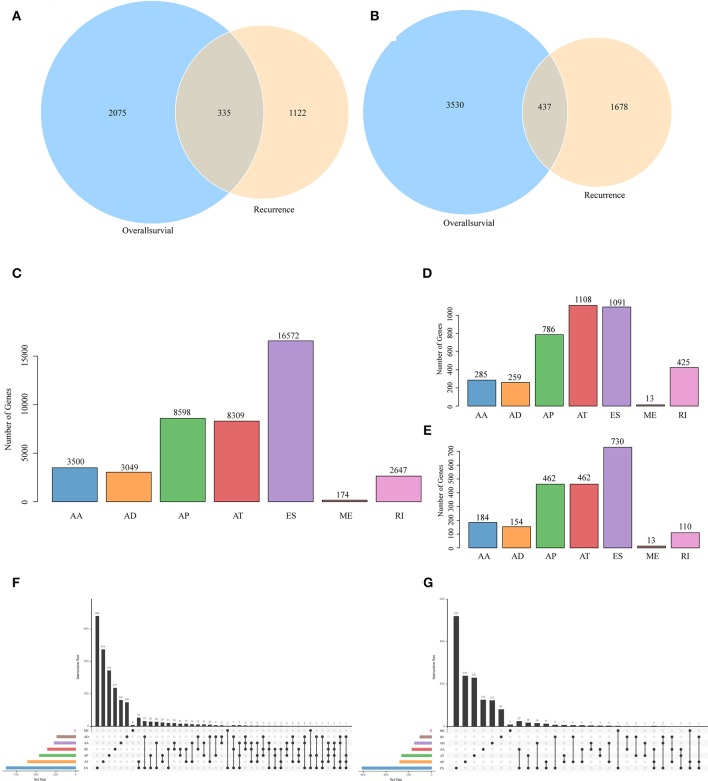
Venn diagram of AS events **(A)** and involved genes **(B)** that were significantly correlated with OS and recurrence. **(C)** Distribution of AS events of seven types. **(D)** Distribution of OS-associated AS events of seven types. **(E)** Distribution of recurrence-associated AS events of seven types. The UpSet intersection diagram shows seven types of OS **(F)**—and recurrence **(G)**—associated AS events in HNSCC. One gene may have up to four types of alternative splicing associated with patient survival or recurrence.

All the AS events in 498 cancer samples were calculated, as shown in Table S1, and the seven AS patterns are presented in Figure 1B, which cover 42849 AS events, including 10,123 genes. The distribution of the seven types of AS events is shown in Figure 2C; as can be seen, one gene might be associated with several types of mRNA AS events. Typically, ES was the main type, accounting for almost 1/3 of all AS events.

### Screening of Prognosis-Related AS Events

To observe the relationships between AS and disease prognosis, all clinical follow-up data for the diseases were integrated to form Table S2, and univariate survival analysis was performed on 42849 AS events to examine the relationships between these AS events and the prognosis for HNSCC patients. When selecting *p* < 0.05, a total of 3697 AS events that were remarkably correlated with survival, involving 2,410 genes, were obtained; in addition, 2115 AS events that were markedly correlated with disease recurrence, covering 1,457 genes, were acquired, as displayed in Table S3. Besides, there were 335 intersections between AS events that were significantly correlated with overall survival and those that were markedly related to recurrence, as presented in Figure 2A. Among them, there were 473 intersected genes among all the involved genes, as shown in Figure 2B, suggesting great consistency between genes involved in overall survival and recurrence-related genes. The numbers of AS events that were markedly correlated with overall survival are plotted in Figure 2D, from which it could be seen that the frequency of overall survival-related ES events was evidently reduced, while the AP and AT events were increased. Besides, among the recurrence related AS events, AT events took up the greatest proportion (Figure 2E), suggesting that most ES events were not correlated with prognosis, while about 10% of AT events were markedly correlated with survival. Furthermore, type analysis was performed on the prognosis-related-gene-selective AS events. Typically, the prognosis-related AS events were selected, and the gene distribution among those genes was calculated (Figures 2F,G). It can be seen from the figure that one gene might be associated with multiple AS events of various types, and these different AS events might be associated with prognosis.

### Analysis of the Prognosis Factors of HNSCC AS Events

**Figure 3:**
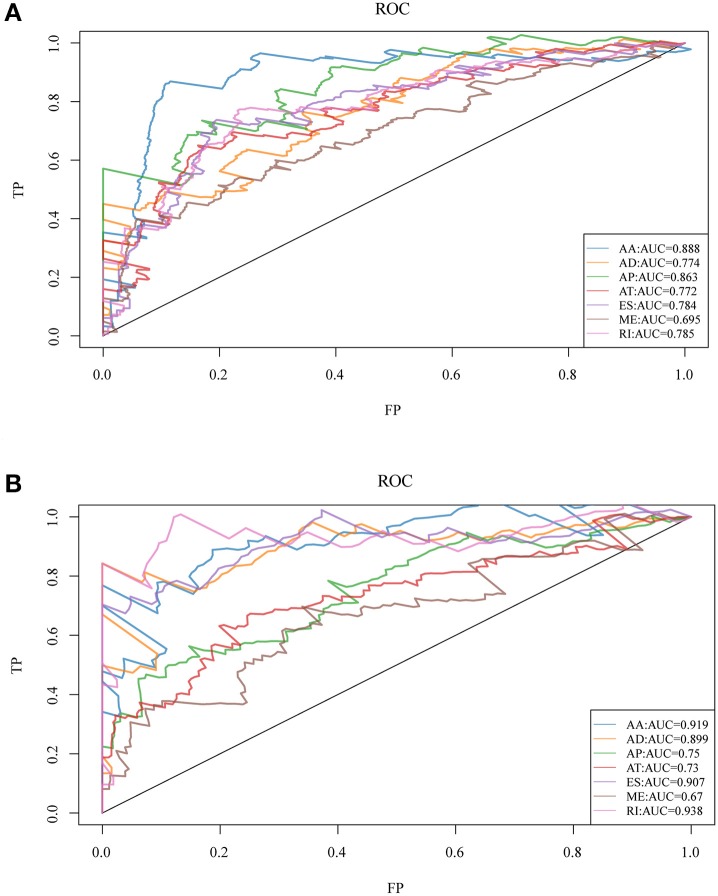
ROC curves for OS **(A)** and recurrence **(B)**-associated AS events of each type for HNSCC patients.

To observe whether the selective AS events could be used as prognosis factors, the 20 most significant genes of each AS type were selected from all prognosis-related AS events for multivariate regression model analysis. Similarly, the significant correlation between AS and genes was observed and the 20 most significant genes were also selected for multivariate regression model analysis so as to observe their classification of prognosis. As could be seen from Figure 3, the seven types of AS events had large areas under the curve (AUC) for prognosis classification, among which, the AA type AS displayed the best overall survival, while the AA and ES types had the best performance among AS significantly correlated with recurrence. Moreover, the top 20 most significant genes were selected from each type of AS event, and the forest maps of these genes are presented in Figures S1, S2. It can be seen that there were 16 genes in the AA splicing type with a hazard ratio (HR) of <1, and four with HR of >1; consistently, there were 16 genes in recurrence-related splicing with a HR of <1 and four with HR of >1. These findings revealed that AS might serve as a new classification method for HNSCC.

### Analysis of Gene Functions in Various Types of AS Events That Were Markedly Correlated With Prognosis

**Figure 4:**
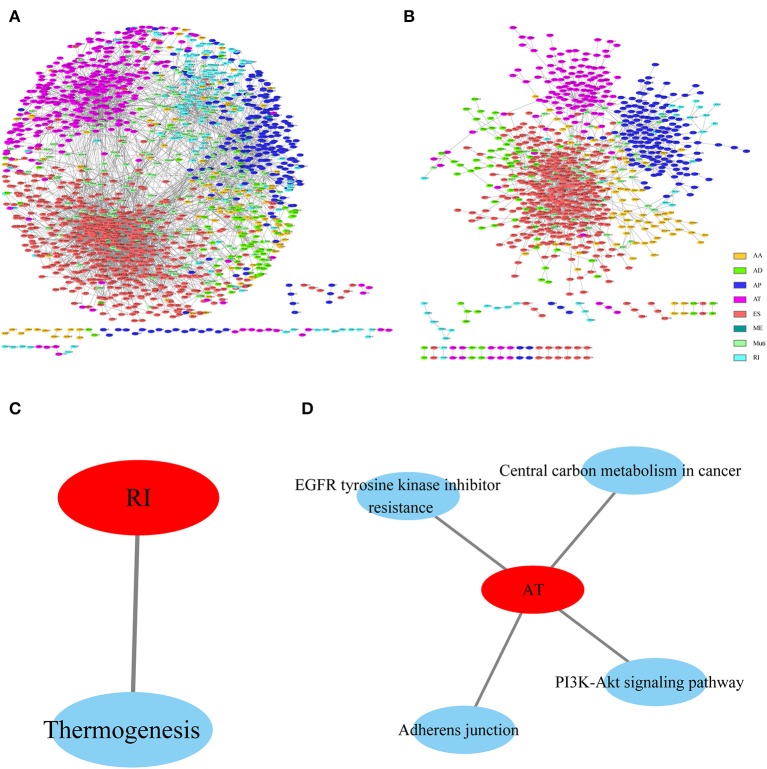
Interaction network of genes with OS **(A)**- and recurrence **(B)**-associated AS events for HNSCC, created by Cytoscape. KEGG enrichment analysis of genes with OS **(C)**- and recurrence **(D)**-associated AS events.

To observe the gene associations among different types of AS events that were apparently correlated with prognosis, these genes were mapped to the String database using a score of >0.4. The gene interactions were obtained to construct the gene interaction network among the various types of AS events that were markedly correlated with prognosis, and Cytoscope was used for visualization. As can be observed from Figures 4A,B, AP and AT displayed most interactions, and most genes in the prognosis-related AS events were associated with protein interactions, revealing that most of these genes were involved in different biological functions. Furthermore, to observe gene function in various types of AS events that were significantly correlated with prognosis, KEGG enrichment analysis was performed on the AS genes in each type that were significantly correlated with prognosis. The results are shown in Figures 4C,D, from which it can be seen that these genes were enriched in multiple disease-related pathways, suggesting that these genes were involved in numerous biological functions.

### Relationships Between Gene Expression Profile and Prognosis in AS Events That Were Markedly Correlated With Prognosis

To observe the relationships between gene expression and prognosis in AS events that were notably correlated with prognosis, TCGA RNA-Seq expression profile data were employed for univariate survival analysis on each gene. Finally, it was discovered that among the 2,410 overall survival-related AS genes, the expression of 399 genes was related to overall survival; whereas, among the 1,457 recurrence-related AS genes, the expression of 194 genes was significantly correlated with recurrence. Furthermore, correlation analysis was performed between these 399 overall-survival genes and the corresponding AS events using the Pearson correlation coefficient. A total of 221 genes (55.39%) were found to be significantly correlated with AS (*P* < 0.05), indicating that the AS events of most genes were associated with their expression. Besides, of the 194 recurrence-related genes, 89 were markedly correlated with AS (45.88%), demonstrating that the AS events of most genes were markedly associated with their expression.

### Selection of HNSCC Feature Genes and Construction of a Prognosis Model

**Figure 5:**
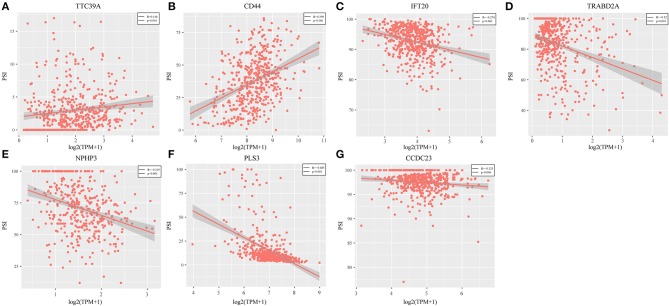
Seven intersected genes related to survival and recurrence were selected. It could be observed TTC39A **(A)** and CD44 **(B)** were positively correlated. But IFT20 **(C)**, TRABD2A **(D)**, NPHP3 **(E)**, PLS3 **(F)**, and CCDC23 **(G)** were negatively correlated (*P* < 0.05).

**Figure 6:**
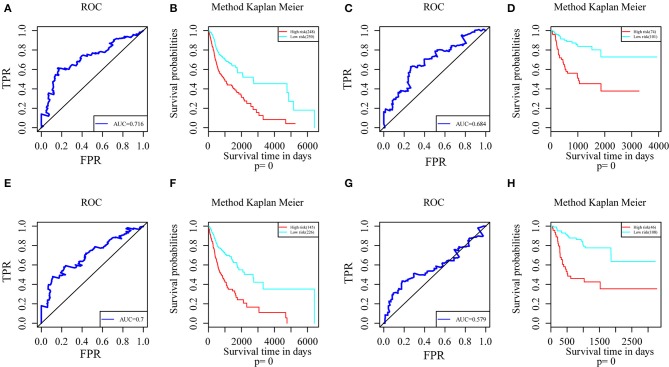
ROC curves and Kaplan-Meier plots of prognostic predictors for seven potential feature genes in HNSCC patients. **(A)** ROC curves with overall survival AUCs of prognostic predictors plotted for transcriptional events in HNSCC. **(B)** Kaplan-Meier curves with overall survival of prognostic predictor plotted for transcriptional events in HNSCC. **(C)** ROC curves with overall survival AUCs of prognostic predictors plotted for AS events in HNSCC. **(D)** Kaplan-Meier curves with overall survival of prognostic predictor plotted for AS events in HNSCC. **(E)** ROC curves with recurrence AUCs of prognostic predictors plotted for AS events in HNSCC. **(F)** Kaplan-Meier curves with recurrence of prognostic predictor plotted for AS events in HNSCC. **(G)** ROC curves with recurrence AUCs of prognostic predictors plotted for AS events in HNSCC. **(H)** Kaplan-Meier curves with recurrence of prognostic predictor plotted for AS events in HNSCC.

Genes with a Pearson correlation coefficient between gene expression and AS events of >0.1 or <-0.1 were selected, comprising 199 survival-related genes and 46 recurrence-related ones. Of the 199 overall survival-related genes, 14 were related to recurrence (CCDC23, TTC39A, CTBS, CD44, TAF1D, CCDC84, DHRS12, IFT20, TRABD2A, SPATS2L, CPNE1, NPHP3, PLS3, and LAMP2). Further multivariate Cox analysis was performed on these 14 genes, and it was found that four genes were related to overall survival (CDDC23, NPHP3, PLS3, and IFT20), while six were related to recurrence (NPHP3, TRABD2A, IFT20, TTC39A, CD44, and PLS3). Finally, seven intersecting genes related to both survival and recurrence were selected; their correlations with transcriptome level are shown in Figure 5. It can be observed from the figure that five genes were negatively correlated. Of these, NPHP3 mutation would lead to puberty nephropathy, retinal degeneration, and liver cirrhosis (39), IFT20 has been reported to be related to lung cancer (40), CD44 has been used as a cancer diagnostic marker (41), and PLS3 has also been used as a biomarker to monitor disease progression (42), indicating that most of these genes played important roles in cancer. Besides, to construct prognosis-predicting indexes that were suitable for HNSCC patients and to facilitate clinical practice, these seven feature genes were used to construct a multivariate survival model so as to observe the classification of prognosis by these seven feature genes at AS event and expression profile levels. As displayed in Figure 6, these seven genes presented favorable prognosis classification effects in both datasets, with a large AUC, indicating that these seven genes might serve as prognosis markers of HNSCC.

## Discussion

Cancer results from genetic and epigenetic changes that interfere with basic mechanisms of the normal cell life cycle, such as DNA repair, replication control, and Cell death (43, 44). In the past decade, the relationship between AS and human diseases has been gradually revealed. Changes in the proportions of splicing isomers of certain key proteins are involved in the occurrence and development of cellular case processes. In the development of disease, although synonymous mutation does not cause a change in the encoded protein sequence, it may change the splicing enhancer or silencer of the exons or introns, thereby affecting the splicing process. Similarly, the RNA terminator appears early due to some mutations. Not only does the protein encoded by this RNA have no function, it may also directly affect the insight process of other normal RNAs and cause diseases. Mutations in cis-acting components cause splicing-related diseases such as Parkinson's frontotemporal dementia (FTDP), muscular dystrophy (MD), and TAU disease represented by myoatrophy. Mutations that occur in trans-acting elements can modulate proteins that control the process of insight and cause disease. If DNA cannot be repaired correctly and effectively after replication or damage, it will lead to gene mutations. Gene mutations occurring at regulatory points will affect the transcriptome even further, resulting in differential proteins that activate or inhibit key functions, leading to infinite proliferation. In all steps of gene expression, AS may provide the greatest potential for molecular diversity and controlled regulation in cells (45). Various molecular complexes composed of RBP, structural RNA, and other protein factors bind to pre-mRNA at various locations (the RNA binding motif) and mediate (46). AS occurs through the expression of complexes on the pre-mRNA regulatory sequence or changes in the gene sequence. AS is not only an important mechanism in the normal cell cycle but is also a key mechanism for the occurrence of gene mutations leading to various pathologies, including tumors. Therefore, identification and analysis of AS are important to advance our understanding of tumor biology.

Head and neck cancers include tumors in head and neck tissues or organs except for the eye, brain, ear, thyroid, and esophagus, and over 90% of head and neck cancers are squamous cell carcinomas (47). The world has witnessed annual totals of about 600,000 new head and neck tumor cases and over 300,000 deaths (48). Head and neck cancer ranks top among systemic tumors in terms of the diversities of its primary site and pathological type. Unfortunately, the survival rate of head and neck cancer patients is low due to the lack of effective risk assessment and means of early diagnosis (49). Head and neck cancer is a highly complicated and heterogeneous disease. Tumor heterogeneity has mediated multiple clinical subtypes of head and neck cancer, which possesses different sensitivity and prognosis to chemotherapeutics and targeted preparations (50). The investigation and clinical application of biomarkers is the key to prognosis assessment, molecular classification, grade determination, recurrence assessment, and early selection of appropriate therapeutics and means. According to the literature, the abnormal splicing level of the *GSN* gene is remarkably higher in tumor tissues than in para-carcinoma tissues and regulates the proliferation process of the HNSCC cell line (51). Meanwhile, the oncogene *DOCK5* variant plays a critical role in the human papilloma virus (HPV)-negative HNSCC and is involved in tumor proliferation, migration, and invasion (52). Moreover, it is also reported that the AS of the *CD44* (53), *LAMA3, DST* (54), and *ESRP* genes has similar effects. Taken together, these results confirm that AS plays an important part in HNSCC genesis and development, which prompted us to systemically analyze the AS time in HNSCC clinical samples and dig out the potential prognosis markers and therapeutic targets.

At present, few complete gene studies on HNSCC AS have been published. A study aimed at identifying AS events (ASE) in human papillomavirus (HPV)-negative HNSCC demonstrated that the analysis of ASE in HPV-negative HNSCC identified multiple changes that may be associated with carcinogenesis, including carcinogenic DOCK5 variants (52). A recent study conducting a genome-wide analysis of AS events using RNA-seq data from the TCGA program in a sample of 464 HNSC patients revealed new AS events associated with carcinogenesis and the immune microenvironment (55). Likewise, another study systematically analyzed the RNA binding protein (RBP) gene mutations, copy number, and gene expression pattern, and changes of AS in these tumors, and AS sequence enrichment and the motif of the change in cancer RBP expression (56). However, screening studies related to survival and recurrence have not been widely studied in HNSCC through the selection of characteristic genes and the establishment of prognostic models.

This paper systemically analyzed 555 HNSCC samples from the TCGA database and mined the relationships between AS events and prognosis. Meanwhile, the key genes affecting the prognosis of HNSCC were also analyzed and mined based on the genome-wide selective splicing events, and the HNSCC samples were classified into high or low risk using the prognosis model constructed based on the gene expression profiles and AS events. Our results verified that AS events can serve as prognosis-predicting factors and potential therapeutic targets under a large sample size, a finding that contributes to the more accurate guidance of clinical treatment and judgment of prognosis. As a consequence, seven potential feature genes were mined, namely *NPHP3, TRABD2A, IFT20, TTC39A, CD44, PLS3*, and *CDDC23*. Additionally, multivariate survival model analysis suggested that these seven feature genes could classify the prognosis of HNSCC well at the AS time and gene expression levels.

Finally, despite the limited ability to detect individual events, we demonstrated that associated AS events can be used to construct high-performance prognostic indicators for HNSCC risk stratification, which is promising in clinical practice. In addition, we found excellent splicing-related networks. These results will be most valuable for deciphering the functional contribution of RNA splicing in HNSCC tumorigenesis. In-depth analysis of RNA splicing patterns may indeed reveal new cancer drivers and provide insights into the mechanisms of these pathways.

## Data Availability Statement

The datasets generated for this study can be found in the TCGA.

## Ethics Statement

Informed consent was obtained from all the patients before surgery, and all experiments were conducted by following the bioethics rules issued by the Research Ethics Committee of Central South University, Changsha, China.

## Author Contributions

YT and XZ designed research. ZL and XC analyzed data. JL and TW statistical data. CC and GLin in functional analysis. GC and YL wrote and modified the paper. MW, GZ, and GLiu prepared all the figures. JW modified the language in the revisions. All authors have read and approved the final manuscript.

### Conflict of Interest

The authors declare that the research was conducted in the absence of any commercial or financial relationships that could be construed as a potential conflict of interest.

## Abbreviations

AS: Alternative splicing
OS: Overall survival
HNSCC: head and neck squamous cell carcinoma
ES: Exon Skip
ME: Mutually Exclusive Exons
RI: Retained Intron
AP: Alternate Promoter
AT: Alternate Terminator
AD: Alternate Donor site
AA: Alternate Acceptor site
AUC: areas under the curve
HR: hazard ratio
TPR: Ture Positive Rate
FPR: False Positive Rate.

## References

[1] Hung YH Liu SA Wang CC Wang CP Jiang RS Wu SH. Treatment outcomes of unknown primary squamous cell carcinoma of the head and neck. PLoS ONE. (2018) 13:e0205365. 10.1371/journal.pone.0205365 30335795 PMC6193660

[2] Chan JYK Zhen G Agrawal N. The role of tumor DNA as a diagnostic tool for head and neck squamous cell carcinoma. Sem Cancer Biol. (2019) 55:1–7. 10.1016/j.semcancer.2018.07.008 30082187

[3] Sannigrahi MK Sharma R Panda NK Khullar M. Role of non-coding RNAs in head and neck squamous cell carcinoma: a narrative review. Oral Dis. (2018) 24:1417–27. 10.1111/odi.12782 28941018

[4] Talmi YP Takes RP Alon EE Nixon IJ Lopez F de Bree R. Prognostic value of lymph node ratio in head and neck squamous cell carcinoma. Head Neck. (2018) 40:1082–90. 10.1002/hed.25080 29394461

[5] Manohar PM Sapir E Bellile E Swiecicki PL Pearson AT Prince ME. Capecitabine after surgical salvage in recurrent squamous cell carcinoma of head and neck. Otolaryngol Head Neck Surg. (2017) 157:995–7. 10.1177/0194599817722948 28809131

[6] Grossberg AJ Mohamed ASR Elhalawani H Bennett WC Smith KE Nolan TS Imaging and clinical data archive for head and neck squamous cell carcinoma patients treated with radiotherapy. Scient Data. (2018) 5:180173 10.1038/sdata.2018.173 PMC6190723 30179230

[7] Karabajakian A Gau M Reverdy T Neidhardt EM Fayette J. Induction chemotherapy in head and neck squamous cell carcinoma: a question of belief. Cancers. (2018) 11:15. 10.3390/cancers11010015 30583519 PMC6357133

[8] Hamilton SN Tran E Berthelet E Wu J Olson R. Early (90-day) mortality after radical radiotherapy for head and neck squamous cell carcinoma: a population-based analysis. Head Neck. (2018) 40:2432–40. 10.1002/hed.25352 30295975

[9] Tomczak K Czerwinska P Wiznerowicz M. The cancer genome atlas (TCGA): an immeasurable source of knowledge. Contemp Oncol. (2015) 19:A68–77. 10.5114/wo.2014.47136 25691825 PMC4322527

[10] Kahles A Lehmann KV Toussaint NC Huser M Stark SG Sachsenberg T. Comprehensive analysis of alternative splicing across tumors from 8,705 patients. Cancer Cell. (2018) 34:211–24.e6. 10.1016/j.ccell.2018.07.001 30078747 PMC9844097

[11] Wei L Jin Z Yang S Xu Y Zhu Y Ji Y. TCGA-assembler 2: software pipeline for retrieval and processing of TCGA/CPTAC data. Bioinformatics. (2018) 34:1615–7. 10.1093/bioinformatics/btx812 29272348 PMC5925773

[12] Nilsen TW Graveley BR. Expansion of the eukaryotic proteome by alternative splicing. Nature. (2010) 463:457–63. 10.1038/nature08909 20110989 PMC3443858

[13] Wang ET Sandberg R Luo S Khrebtukova I Zhang L Mayr C. Alternative isoform regulation in human tissue transcriptomes. Nature. (2008) 456:470–6. 10.1038/nature07509 18978772 PMC2593745

[14] Salton M Misteli T. Small molecule modulators of Pre-mRNA splicing in cancer therapy. Trends Mol Med. (2016) 22:28–37. 10.1016/j.molmed.2015.11.005 26700537 PMC4707101

[15] Li Y Sun N Lu Z Sun S Huang J Chen Z. Prognostic alternative mRNA splicing signature in non-small cell lung cancer. Cancer Lett. (2017) 393:40–51. 10.1016/j.canlet.2017.02.016 28223168

[16] Komeno Y Huang YJ Qiu J Lin L Xu Y Zhou Y. SRSF2 is essential for hematopoiesis, and its myelodysplastic syndrome-related mutations dysregulate alternative pre-mRNA splicing. Mol Cell Biol. (2015) 35:3071–82. 10.1128/MCB.00202-15 26124281 PMC4525309

[17] Su C-H Dhananjaya D Tarn WY. Alternative splicing in neurogenesis and brain development. Front Mol Biosci. (2018) 5:12. 10.3389/fmolb.2018.00012 29484299 PMC5816070

[18] Van Alstyne M Simon CM Sardi SP Shihabuddin LS Mentis GZ Pellizzoni L. Dysregulation of Mdm2 and Mdm4 alternative splicing underlies motor neuron death in spinal muscular atrophy. Genes Dev. (2018) 32:1045–59. 10.1101/gad.316059.118 30012555 PMC6075148

[19] Bartel F Taubert H Harris LC. Alternative and aberrant splicing of MDM2 mRNA in human cancer. Cancer Cell. (2002) 2:9–15. 10.1016/S1535-6108(02)00091-0 12150820

[20] Martinez-Montiel N Rosas-Murrieta NH Anaya Ruiz M Monjaraz-Guzman E Martinez-Contreras R. Alternative splicing as a target for cancer treatment. Int J Mol Sci. (2018) 19:545. 10.3390/ijms19020545 29439487 PMC5855767

[21] Oltean S Bates DO. Hallmarks of alternative splicing in cancer. Oncogene. (2014) 33:5311–8. 10.1038/onc.2013.533 24336324

[22] Singh R Gupta SC Peng WX Zhou N Pochampally R Atfi A. Regulation of alternative splicing of Bcl-x by BC200 contributes to breast cancer pathogenesis. Cell Death Dis. (2016) 7:e2262. 10.1038/cddis.2016.168 27277684 PMC5143396

[23] Hamdollah Zadeh MA Amin EM Hoareau-Aveilla C Domingo E Symonds KE Ye X. Alternative splicing of TIA-1 in human colon cancer regulates VEGF isoform expression, angiogenesis, tumour growth and bevacizumab resistance. Mol Oncol. (2015) 9:167–78. 10.1016/j.molonc.2014.07.017 25224594 PMC4286123

[24] Marzese DM Manughian-Peter AO Orozco JIJ Hoon DSB. Alternative splicing and cancer metastasis: prognostic and therapeutic applications. Clin Exp Metast. (2018) 35:393–402. 10.1007/s10585-018-9905-y 29845349

[25] Ghigna C Giordano S Shen H Benvenuto F Castiglioni F Comoglio PM. Cell motility is controlled by SF2/ASF through alternative splicing of the Ron protooncogene. Mol Cell. (2005) 20:881–90. 10.1016/j.molcel.2005.10.026 16364913

[26] Sotillo E Barrett DM Black KL Bagashev A Oldridge D Wu G. Convergence of acquired mutations and alternative splicing of CD19 enables resistance to CART-19 immunotherapy. Cancer Disc. (2015) 5:1282–95. 10.1158/2159-8290.CD-15-1020 26516065 PMC4670800

[27] Kong-Beltran M Seshagiri S Zha J Zhu W Bhawe K Mendoza N. Somatic mutations lead to an oncogenic deletion of met in lung cancer. Cancer Res. (2006) 66:283–9. 10.1158/0008-5472.CAN-05-2749 16397241

[28] Zhu J Chen Z Yong L. Systematic profiling of alternative splicing signature reveals prognostic predictor for ovarian cancer. Gynecol Oncol. (2018) 148:368–74. 10.1016/j.ygyno.2017.11.028 29191436

[29] Bjorklund SS Panda A Kumar S Seiler M Robinson D Gheeya J. Widespread alternative exon usage in clinically distinct subtypes of Invasive Ductal Carcinoma. Scient Rep. (2017) 7:5568. 10.1038/s41598-017-05537-0 28717182 PMC5514065

[30] Robertson AG Shih J Yau C Gibb EA Oba J Mungall KL. Integrative analysis identifies four molecular and clinical subsets in uveal melanoma. Cancer Cell. (2017) 32:204–20.e15. 10.1016/j.ccell.2017.07.003 28810145 PMC5619925

[31] Marcelino Meliso F Hubert CG Favoretto Galante PA Penalva LO. RNA processing as an alternative route to attack glioblastoma. Hum Genet. (2017) 136:1129–41. 10.1007/s00439-017-1819-2 28608251 PMC6685205

[32] Ryan M Wong WC Brown R Akbani R Su X Broom B. TCGASpliceSeq a compendium of alternative mRNA splicing in cancer. Nucleic Acids Res. (2016) 44:D1018–22. 10.1093/nar/gkv1288 26602693 PMC4702910

[33] Cen WN Pang JS Huang JC Hou JY Bao WG He RQ. The expression and biological information analysis of miR-375-3p in head and neck squamous cell carcinoma based on 1825 samples from GEO, TCGA, and peer-reviewed publications. Pathol Res Practice. (2018) 214:1835–47. 10.1016/j.prp.2018.09.010 30243807

[34] Tugizimana F Steenkamp PA Piater LA Dubery IA. A conversation on data mining strategies in LC-MS untargeted metabolomics: pre-processing and pre-treatment steps. Metabolites. (2016) 6:40. 10.3390/metabo6040040 27827887 PMC5192446

[35] Ronneberg TA Freeland SJ Landweber LF. Genview and gencode: a pair of programs to test theories of genetic code evolution. Bioinformatics. (2001) 17:280–1. 10.1093/bioinformatics/17.3.280 11294793

[36] Ryan MC Cleland J Kim R Wong WC Weinstein JN. SpliceSeq: a resource for analysis and visualization of RNA-Seq data on alternative splicing and its functional impacts. Bioinformatics. (2012) 28:2385–7. 10.1093/bioinformatics/bts452 22820202 PMC3436850

[37] O'Quigley J Moreau T. Cox's regression model: computing a goodness of fit statistic. Comp Methods Programs Biomed. (1986) 22:253–6. 10.1016/0169-2607(86)90001-5 3524984

[38] Szklarczyk D Morris JH Cook H Kuhn M Wyder S Simonovic M. The STRING database in 2017: quality-controlled protein-protein association networks, made broadly accessible. Nucleic Acids Res. (2017) 45:D362–d8. 10.1093/nar/gkw937 27924014 PMC5210637

[39] Olbrich H Fliegauf M Hoefele J Kispert A Otto E Volz A. Mutations in a novel gene, NPHP3, cause adolescent nephronophthisis, tapeto-retinal degeneration and hepatic fibrosis. Nat Genet. (2003) 34:455–9. 10.1038/ng1216 12872122

[40] Dong YZ Meng XM Li GS. Long non-coding RNA SNHG15 indicates poor prognosis of non-small cell lung cancer and promotes cell proliferation and invasion. Europ Rev Med Pharmacol Sci. (2018) 22:2671–9. 10.26355/eurrev_201805_14963 29771418

[41] Matsumura Y Tarin D. Significance of CD44 gene products for cancer diagnosis and disease evaluation. Lancet. (1992) 340:1053–8. 10.1016/0140-6736(92)93077-Z 1357452

[42] Tang N Gibson H Germeroth T Porcu P Lim HW Wong HK. T-plastin (PLS3) gene expression differentiates Sezary syndrome from mycosis fungoides and inflammatory skin diseases and can serve as a biomarker to monitor disease progression. Br J Dermatol. (2010) 162:463–6. 10.1111/j.1365-2133.2009.09587.x 19995369 PMC3928081

[43] Feinberg AP Koldobskiy MA Gondor A. Epigenetic modulators, modifiers and mediators in cancer aetiology and progression. Nat Rev Genetics. (2016) 17:284–99. 10.1038/nrg.2016.13 26972587 PMC4888057

[44] Hanahan D Weinberg RA. Hallmarks of cancer: the next generation. Cell. (2011) 144:646–74. 10.1016/j.cell.2011.02.013 21376230

[45] Fu XD Ares M Jr. Context-dependent control of alternative splicing by RNA-binding proteins. Nat Rev Genet. (2014) 15:689–701. 10.1038/nrg3778 25112293 PMC4440546

[46] Siegfried Z Karni R. The role of alternative splicing in cancer drug resistance. Curr Opin Genet Dev. (2018) 48:16–21. 10.1016/j.gde.2017.10.001 29080552

[47] Orlandi E Alfieri S Simon C Trama A Licitra L. Treatment challenges in and outside a network setting: head and neck cancers. Europ J Surg Oncol. (2019) 45:40–5. 10.1016/j.ejso.2018.03.012 29478741

[48] Maghami E Koyfman SA Weiss J. Personalizing postoperative treatment of head and neck cancers. Am Soc Clin Oncol Educ Book. (2018) 38:515–22. 10.1200/EDBK_201087 30231315

[49] Wierzbicka M Napierala J. Updated national comprehensive cancer network guidelines for treatment of head and neck cancers 2010–2017. Otolaryngol Pol. (2017) 71:1–6. 10.5604/01.3001.0010.7193 29327681

[50] Shang W Zhang Q Huang Y Shanti R Alawi F Le A. Cellular plasticity-targeted therapy in head and neck cancers. J Dental Res. (2018) 97:654–64. 10.1177/0022034518756351 29486673

[51] Kelley DZ Flam EL Guo T Danilova LV Zamuner FT Bohrson C. Functional characterization of alternatively spliced GSN in head and neck squamous cell carcinoma. Transl Res. (2018) 202:109–19. 10.1016/j.trsl.2018.07.007 30118659 PMC6218276

[52] Liu C Guo T Xu G Sakai A Ren S Fukusumi T. Characterization of alternative splicing events in HPV-negative head and neck squamous cell carcinoma identifies an oncogenic DOCK5 variant. Clin Cancer Res. (2018) 24:5123–32. 10.1158/1078-0432.CCR-18-0752 29945995 PMC6440699

[53] Wang SJ Wong G de Heer AM Xia W Bourguignon LY. CD44 variant isoforms in head and neck squamous cell carcinoma progression. Laryngoscope. (2009) 119:1518–30. 10.1002/lary.20506 19507218 PMC2718060

[54] Li R Ochs MF Ahn SM Hennessey P Tan M Soudry E. Expression microarray analysis reveals alternative splicing of LAMA3 and DST genes in head and neck squamous cell carcinoma. PLoS ONE. (2014) 9:e91263. 10.1371/journal.pone.0091263 24675808 PMC3967989

[55] Li ZX Zheng ZQ Wei ZH Zhang LL Li F Lin L. Comprehensive characterization of the alternative splicing landscape in head and neck squamous cell carcinoma reveals novel events associated with tumorigenesis and the immune microenvironment. Theranostics. (2019) 9:7648–65. 10.7150/thno.36585 31695792 PMC6831462

[56] Dvinge H Bradley RK. Widespread intron retention diversifies most cancer transcriptomes. Genome Med. (2015) 7:45. 10.1186/s13073-015-0168-9 26113877 PMC4480902

