# Supplementary figures and images for: Systemic Analysis of RNA Alternative Splicing Signals Related to the Prognosis for Head and Neck Squamous Cell Carcinoma
Source: Front Oncol. 2020 Feb 7;10:87. doi: 10.3389/fonc.2020.00087 (PMC7025462; doi:10.3389/fonc.2020.00087)

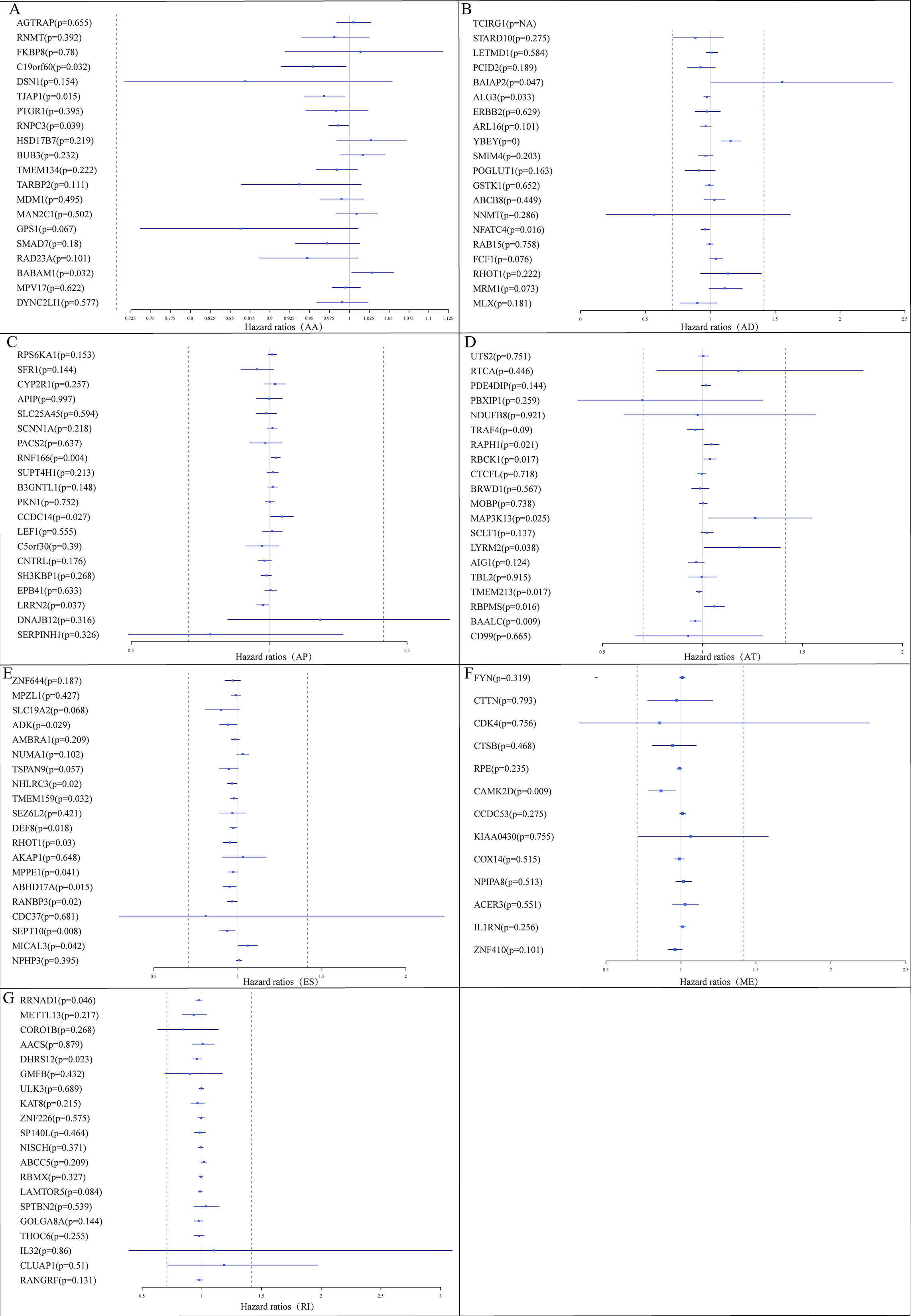

Supplement: Supplementary file 1 [file Image_1.TIF]

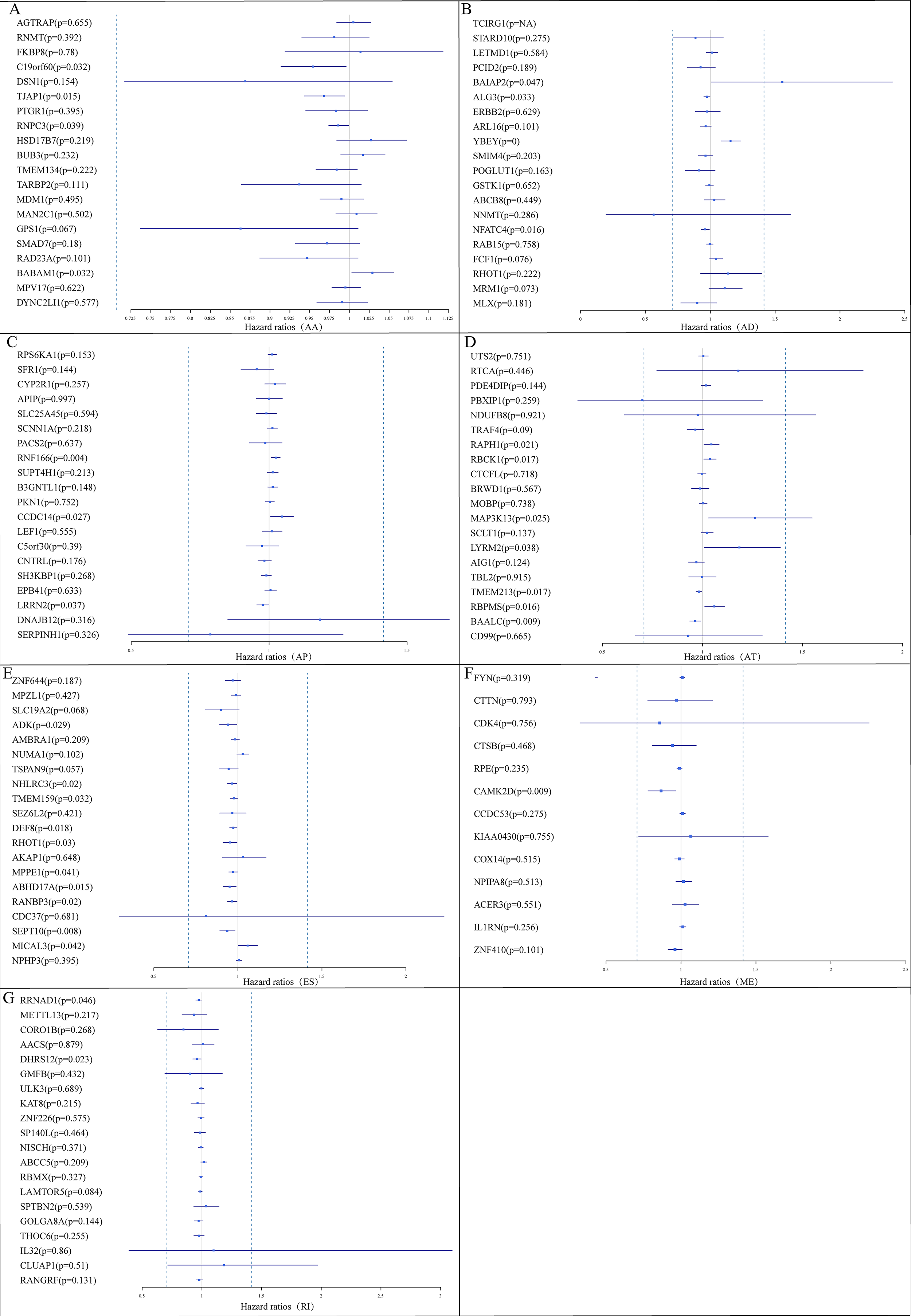

Supplement: Supplementary file 2 [file Image_2.TIF]
