# Supplementary material for: Systemic Analysis of RNA Alternative Splicing Signals Related to the Prognosis for Head and Neck Squamous Cell Carcinoma
Source: Front Oncol. 2020 Feb 7;10:87. doi: 10.3389/fonc.2020.00087 (PMC7025462; doi:10.3389/fonc.2020.00087)
Supplement: Supplementary file 6 [file Data_Sheet_1.docx]

**Figure S1.** Forest plots of hazard ratios of recurrence associated alternative events in HNSCC. (A) Hazard ratios of top 20 genes with recurrence associated AA events. (B) Hazard ratios of top 20 genes with recurrence associated AD events. (C) Hazard ratios of top 20 genes with recurrence associated AP events. (D) Hazard ratios of top 20 genes with recurrence associated AT events. (E) Hazard ratios of top 20 genes with recurrence associated ES events. (F) Hazard ratios of top 13 genes with recurrence associated ME events. (G) Hazard ratios of top 20 genes with recurrence associated RI events.

**Figure S2.** hazard ratios of recurrence associated alternative events by forest plots in HNSCC. (A) Top 20 genes of Hazard ratios with AA events. (B) Top 20 genes of Hazard ratios with AD events. (C) Hazard ratios of top 20 genes with recurrence associated AP events. (D) Hazard ratios of top 20 genes with recurrence associated AT events. (E) Top 20 genes of Hazard ratios with ES events. (F) Top 13 genes of Hazard ratios with ME events. (G) Hazard ratios of top 20 genes with recurrence associated RI events.

**Table S1**. All the AS in 498 cancer samples were calculated.

**Table S2**.All clinical follow-up data for the disease were integrated.

**Table S3**. 2115 AS envents were markedly and 1457 genes were acquired correlated with disease.
